# Guideline-conform statin use reduces overall mortality in patients with compensated liver disease

**DOI:** 10.1038/s41598-019-47943-6

**Published:** 2019-08-12

**Authors:** Lukas W. Unger, Bernadette Forstner, Stephan Schneglberger, Moritz Muckenhuber, Ernst Eigenbauer, David Bauer, Bernhard Scheiner, Mattias Mandorfer, Michael Trauner, Thomas Reiberger

**Affiliations:** 1 Division of General Surgery, Department of Surgery, Medical University of Vienna, Spitalgasse 23 A-1090, Vienna, Austria; 2 IT-Systems & Communications, Medical University of Vienna, Spitalgasse 23 A-1090, Vienna, Austria; 3 Division of Gastroenterology and Hepatology, Department of Internal Medicine III, Medical University of Vienna, Spitalgasse 23 A-1090, Vienna, Austria; 4 Vienna Hepatic Hemodynamic Laboratory, Medical University of Vienna, Spitalgasse 23 A-1090, Vienna, Austria

**Keywords:** Dyslipidaemias, Liver fibrosis

## Abstract

Statins reduce cardiovascular risk. However, “real-life” data on statin use in patients with chronic liver disease and its impact on overall and liver-related survival are limited. Therefore, we assessed 1265 CLD patients stratified as advanced (ACLD) or non-advanced (non-ACLD) stage. Statin indication was evaluated according to the 2013 ACC/AHA guidelines and survival-status was verified by national death registry data. Overall, 122 (9.6%) patients had an indication for statin therapy but did not receive statins, 178 (14.1%) patients were on statins and 965 (76.3%) patients had no indication for statins. Statin underutilization was 34.2% in non-ACLD and 48.2% in ACLD patients. In non-ACLD patients, survival was worse without a statin despite indication as compared to patients on statin or without indication (log-rank p = 0.018). In ACLD patients, statin use did not significantly impact on survival (log-rank p = 0.264). Multivariate cox regression analysis confirmed improved overall survival in patients with statin as compared to patients with indication but no statin (HR 0.225; 95%CI 0.053–0.959; p = 0.044) and a trend towards reduced liver-related mortality (HR 0.088; 95%CI 0.006–1.200; p = 0.068). This was not observed in ACLD patients. In conclusion, guideline-confirm statin use is often withhold from  patients with liver disease and this underutilization is associated with impaired survival in non-ACLD patients.

## Introduction

Obesity and the metabolic syndrome are on the rise^1^ and clearly associated with increased atherosclerotic cardiovascular disease (ASCVD)-related mortality. Dyslipidemia is a major risk factor for ASCVD development and progression^2^ and therefore, 2013 ACC/AHA guidelines recommend lipid-lowering therapy in patients at increased risk for ASCVD^3^. The liver, due to its prominent role in lipid metabolism, is a major modulator of ASCVD risk^4^ as most cholesterol is synthesized endogenously in hepatocytes, while dietary intake of cholesterol is not a major determinant of systemic cholesterol levels^5^. Due to the importance of endogenous cholesterol synthesis, pharmacologic blockade of the 3-hydroxy-3-methylglutaryl-CoA (HMG-CoA) reductase by statins results in a decrease of systemic low-density lipoprotein (LDL) cholesterol levels^6^. In general, statins are well tolerated in most patients, while 10–15% experience adverse events such as myalgia with or without elevation of creatinine kinase (CK)^6,7^. Overall, these potential side effects are outweighed by the positive lipid-lowering and other pleiotropic effects of statins. In addition, statins have been shown to decrease the risk of hepatic decompensation^8,9^. These favorable effects might be explained by amelioration of (sinusoidal) endothelial dysfunction^10,11^, a reduction in hepatic venous pressure gradient (HVPG) and improvement of hepatic function^12^. While simvastatin did not improve hepatic steatosis, necroinflammation and fibrosis on liver biopsy in a small placebo-controlled randomized trial in NAFLD patients^13^, other studies reported several beneficial effects of statins in patients with CLD: Among patients with hepatitis C virus (HCV) infection, statin use decreased the risk of hepatic decompensation, death^14,15^ and HCC) development^16,17^. Similarily, a lower risk for progression to cirrhosis and hepatic decompensation was also observed among hepatitis B virus (HBV) infected patients on statin therapy^18^. In patients with primary biliary cholangitis (PBC), simvastatin and low-dose atorvastatin were safe, decreased dyslipidemia, improved endothelial function and reduced oxidative stress^19,20^. Despite this increasing body of evidence suggesting beneficial effects of statins on liver disease, recent reports suggested an underutilization of statins, at least in the setting of non-alcoholic fatty liver disease (NAFLD)^21^.

While ASCVD mortality is not increased in patients with chronic HBV^22^ and PBC^23^, ASCVD risk is increased in patients with heavy alcohol consumption^24^, HCV^25^ and NAFLD^26^. As a major limitation, however, most studies do not differentiate between patients with and without advanced chronic liver disease (ACLD), although dyslipidemia seems to be affected by severity of liver disease^4^.

Therefore, further data on the efficacy of statin therapy is warranted. Here, we present data on statin utilization rates and effect on overall survival in patients with compensated (i.e. non-ACLD) and ACLD.

## Results

**Figure 1:**
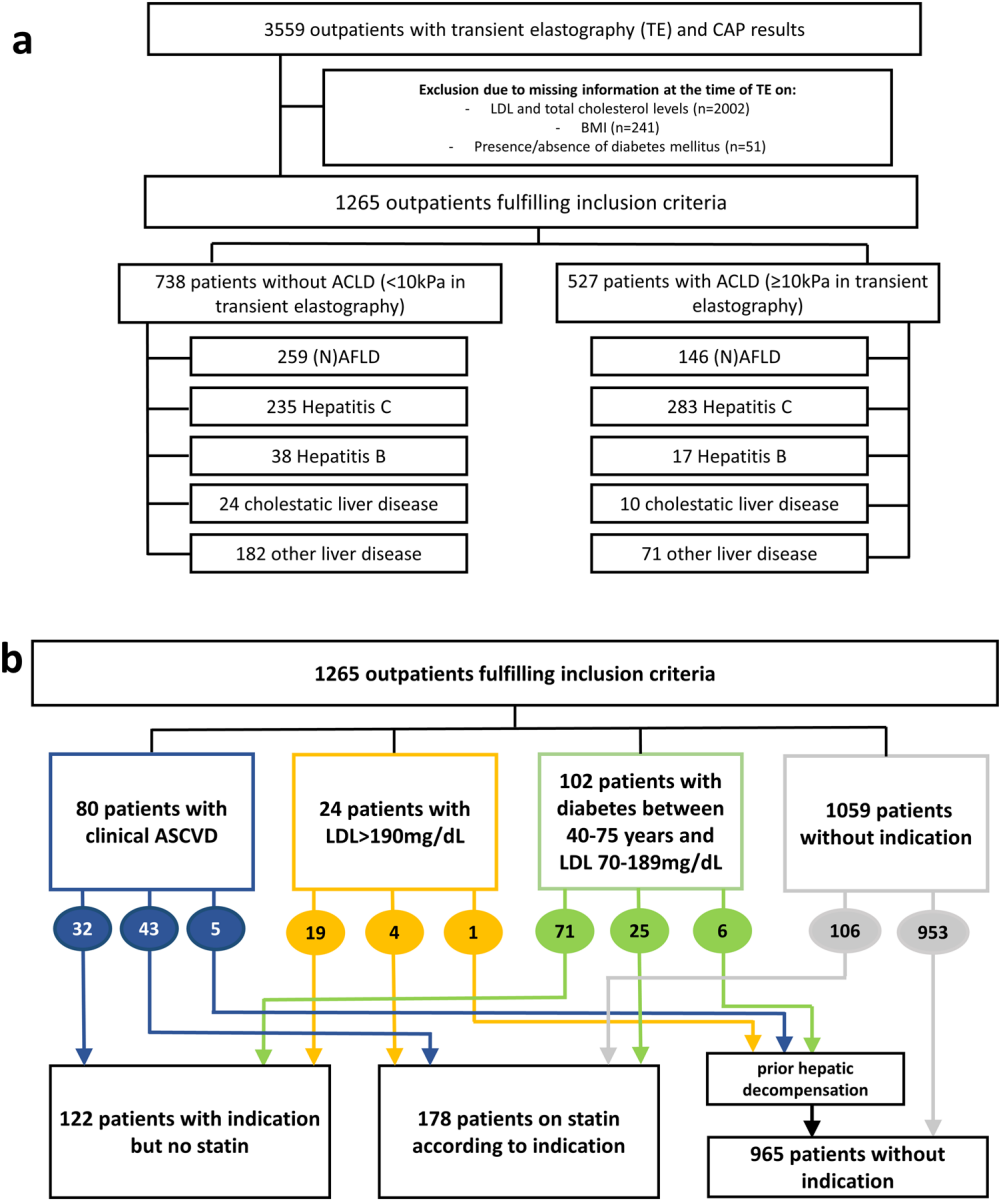
(**a**) Patient flow chart. (**b**) Indications for statin therapy according to the American College of Cardiology (ACC) and the American Heart Association (AHA).

The study population comprised of 1265 consecutive patients with known etiology of liver disease and available data on liver stiffness measurement (LSM). The patient flow chart indicating the number of patients with different liver disease etiologies and their non-ACLD or ACLD status is shown in Fig. 1a.

### Differences in serum lipid levels according to underlying liver disease

**Figure 2:**
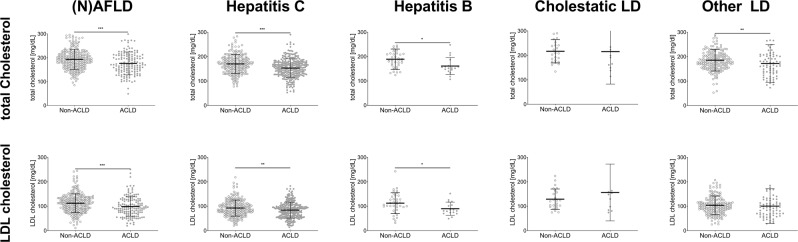
Total cholesterol levels and LDL levels in non-ACLD and ACLD patients. Data is presented as scatter plot for the respective etiology. *** indicates p < 0.001, ** indicates p < 0.01, * indicates p < 0.05.

As the lipid profile is influenced by the underlying liver disease, the difference between patients of the respected subgroups without ACLD (LSM < 10kPa) were compared to patients with ACLD (LSM ≥ 10kPa) (Table 1, Fig. 2). Total cholesterol levels were significantly lower in patients with ACLD compared to non-ACLD patients in all etiologies except for “cholestatic liver diseases” (Fig. 2, upper row panels). LDL cholesterol levels were similarly decreased in ACLD patients except for “cholestatic liver diseases” and “other liver diseases” (Fig. 2, lower row panels). Thus, it seems that total cholesterol and LDL levels decrease when patients progress to ACLD, irrespective of the underlying disease. A detailed outline of total cholesterol and LDL cholesterol levels is presented in Table 1.

**Table 1:** Serum lipid levels in patients with and without ACLD.

Etiology		non-ACLD n = 738	ACLD n = 527	p-value
(N)AFLD	Total-C	191.0 (164.0–217.0)	171.0 (143.0–209.3)	<0.001
	LDL-C	112.0 (86.8–135.2)	93.4 (71.1–125.0)	<0.001
Hepatitis C	Total-C	170.0 (143.0–193.0)	155 (127.0–177.0)	<0.001
	LDL-C	89.8 (71.2–111.0)	83.2 (58.4–104.4)	0.006
Hepatitis B	Total-C	185.5 (162.0–211.5)	153.0 (143.0–174.5)	0.013
	LDL-C	106.4 (90.0–242.8)	80.8 (72.0–151.8)	0.039
Cholestatic liver diseases	Total-C	208.5 (175.3–250.0)	178.0 (146.3–225.8)	0.116
	LDL-C	121.6 (98.1–162.1)	138.7 (83.2–159.5)	0.965
Other liver diseases	Total-C	181.0 (157.8–207.3)	162.0 (130.0–202.0)	0.004
	LDL-C	100.4 (79.5–122.9)	93.0 (65.0–116.6)	0.070

Numerical variables are presented as median (Q1–Q3). Numerical values are presented as [mg/dL]. Total-C = total cholesterol; LDL-C = LDL cholesterol.

### Necessity of statin therapy according to 2013 ACC/AHA guidelines

To evaluate necessity of statin initiation, the 2013 ACC/AHA guidelines were followed which do not differentiate between the underlying liver diseases. In the overall cohort, 21 patients had a history of stroke, 33 of myocardial infarction and 26 of coronary heart disease and therefore, required a statin due to clinical ASCVD. 24 patients had an LDL > 190 mg/dL and required a statin for primary prevention and 102 patients suffered from diabetes (NIDDM or IDDM), were between 40–75 years of age and had LDL levels between 70–189 mg/dL. Of these 206 patients, 12 had hepatic decompensation episodes prior to LSM and had, therefore, a “formal” contraindication for statin initiation. Finally, 72 of the identified 194 patients with an indication for statin therapy were already on a statin, while 122 patients with a clear indication did not receive a statin prescription. Additionally, 106 patients were on statin therapy prior to LSM. Therefore, there was a total of 178 patients on statin therapy.

In summary, we subjected three groups of patients to further analyses: 122 patients with an indication for statin therapy but without established therapy (“no statin despite indication”), 178 patients with statin use (“on statin”) and 965 patients without an indication for statin therapy (“no indication for statin”, see Fig. 1b). Patient characteristics of the respective subgroups (non-ACLD and ACLD) are presented in Table 2.

**Table 2:** Patient characteristics in non-ACLD and ACLD patients.

	No statin despite indication	On statin therapy	No indication for statin	p-value
**non-ACLD**				
# of patients	55	106	577	
Age [years]	55.4 (17.4)	58.0 (14.6)	46.0 (20.68)	<0.001
CAP [dB/m]	252 (98)	274.5 (80.3)	242 (87)	<0.001
BMI [kg/m²]	26.2 (8.4)	27.0 (8.1)	25.2 (6.0)	0.003
Male sex (%)	29 (52.7%)	58 (54.7%)	309 (53.6%)	0.969
Arterial hypertension (%)	30 (54.5%)	77 (72.6%)	116 (20.1%)	<0.001
NIDDM (%)	24 (43.6%)	33 (31.1%)	11 (1.9%)	<0.001
IDDM (%)	3 (5.5%)	6 (5.7%)	12 (2.1%)	
(N)AFLD	23 (41.8%)	55 (51.9%)	181 (31.4%)	<0.001
Cholestatic LD	3 (5.5%)	8 (7.5%)	13 (2.3%)	
Hepatitis C	16 (29.1%)	20 (18.9%)	199 (34.5%)	
Hepatitis B	3 (5.5%)	4 (3.8%)	31 (5.4%)	
Other LD	10 (18.2%)	19 (17.9%)	153 (26.5%)	
**ACLD**				
# of patients	67	72	388	
Age [years]	60.4 (15.0)	60.8 (10.6)	54.3 (14.7)	<0.001
CAP [dB/m]	290 (85)	299 (130)	247 (96)	<0.001
BMI [kg/m²]	29.7 (5.8)	28.4 (7.9)	26.4 (6.5)	<0.001
Male sex (%)	43 (64.2%)	50 (69.4%)	249 (64.2%)	0.685
Arterial hypertension (%)	42 (62.7%)	49 (68.1%)	100 (25.8%)	<0.001
NIDDM (%)	39 (58.2%)	28 (38.9%)	25 (6.4%)	<0.001
IDDM (%)	7 (10.4%)	12 (16.7%)	38 (9.8%)	
(N)AFLD	29 (43.3%)	29 (40.3%)	88 (22.7%)	0.001
Cholestatic LD	0 (0.0%)	1 (1.4%)	9 (2.3%)	
Hepatitis C	31 (46.3%)	29 (40.3%)	223 (57.5%)	
Hepatitis B	0 (0.0%)	5 (6.9%)	12 (3.1%)	
Other LD	7 (10.4%)	8 (11.1%)	56 (14.4%)	

CAP = controlled attenuation parameters; BMI = body-mass index, NIDDM = non-insulin dependent diabetes mellitus; IDDM = insulin-dependent diabetes mellitus; LD = liver disease.

### Underutilization of statin therapy and metabolic comorbidities

Overall, 300/1265 (23.7%) patients presented with dyslipidemia requiring therapy, however, 122/300 (40.7%) did not receive an indicated statin therapy. Interestingly, underutilization rates varied between liver disease etiologies: The rate of statin underutilization was highest in hepatitis C (47/96; 49.0%), “other liver diseases” (17/44; 38.6%) and (N)AFLD (52/136; 38.2%) followed by hepatitis B (3/12; 25.0%) and cholestatic liver disease (3/12; 25.0%).

Additionally to the higher ASCVD risk resulting from dyslipidemia, both groups of patients with “no statin despite indication” and “on statin” had a higher prevalence of diabetes (p < 0.001 for both the ACLD and the non-ACLD setting), and arterial hypertension (p < 0.001 for both the ACLD and the non-ACLD setting) as compared to the”no indication for statin” patient group. This underlines an increased risk for cardiovascular events derived from comorbidities of diabetes and arterial hypertension in the “no statin despite indication” and the “on statin group”. Differences in metabolic comorbidities are depicted in Supplementary Fig. 1.

### Impaired overall survival in patients without guideline-conform statin therapy

**Figure 3:**
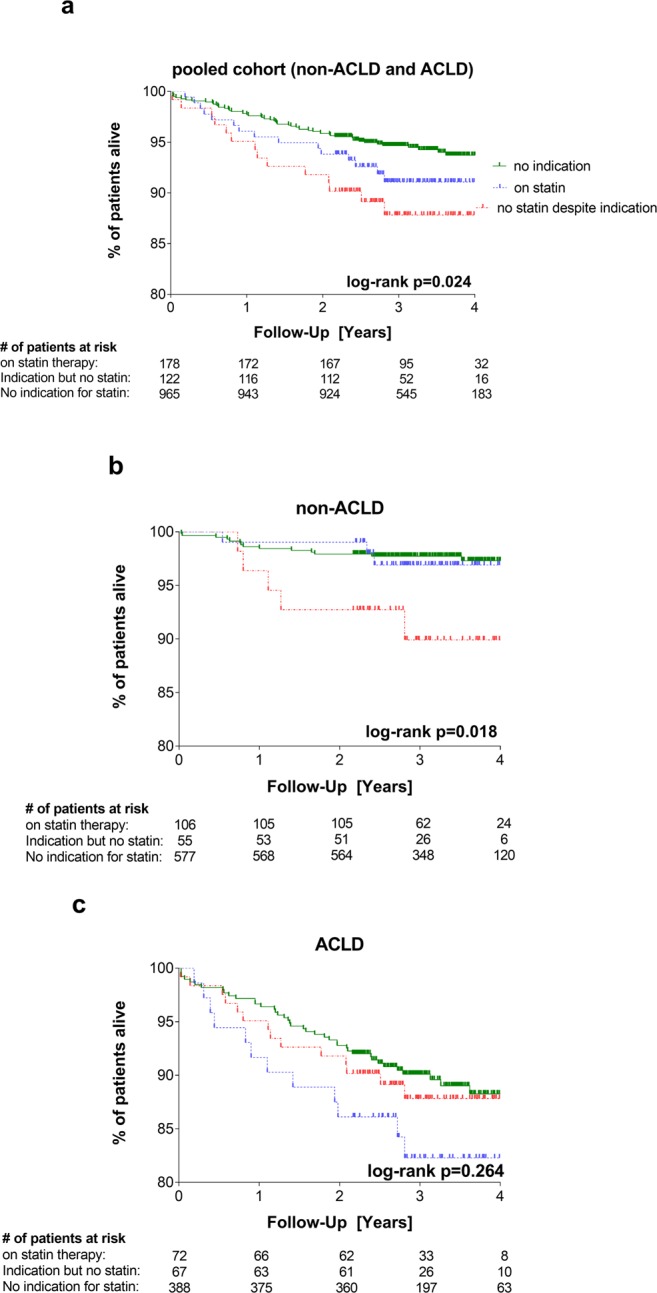
Kaplan Meier survival curves for overall survival. (**a**) Pooled cohort of non-ACLD and ACLD patients. Overall survival was significantly different between groups (p = 0.024) (**b**) non-ACLD patients (LSM < 10kPa). Overall survival was significantly different between groups (log-rank p = 0.018) (**c**) ACLD patients (LSM ≥ 10kPa). Overall survival was not significantly different between groups (log-rank p = 0.264).

Finally, Austrian national death registry data were used to assess survival in the respective subgroups (Fig. 3). While Fig. 3a depicts a pooled analysis on overall survival, patients with ACLD indicating a dismal prognosis per se were analyzed separately from non-ACLD patients in the subsequent analyses. In the pooled cohort, patients without an indication for statin therapy showed the best long-term survival rates while in the “no statin despite indication” group had the worst survival (log-rank p = 0.024).

In the non-ALCD setting, the patient group “no statin despite indication” had worse overall survival rates as compared to patients within the “on statin” and “no indication” groups (Fig. 3b; log-rank p = 0.018): Estimated survival rates after 1, 2, 3 and 4 years were 96.4%, 92.7%, 89.9% and 89.9% in the “no statin despite indication” group, 99.1%, 99.1%, 97.0 and 97.0% in the “on statin” group, and 98.4%, 97.9%, 97.7% and 97.3% in the “no indication” group, respectively.

In the ACLD setting, however, there was no difference in survival between the subgroups (Fig. 3c; log-rank p = 0.264): Estimated survival rates after 1, 2, 3 and 4 years were 94.0%, 91.0%, 86.2% and 86.2% in the “no statin despite indication” group, 91.7%, 86.1%, 82.3% and 82.3% in the “on statin” group, and 96.6%, 92.8%, 90.1% and 88.2% in the “no indication” group, respectively.

Notably, baseline characteristics were different between groups. While we have addressed these differences by adjusting the data analyses in the following multivariate models (Table 3, Table 4), no adjustments could be performed in the log-rank analyses.

**Table 3:** Cox regression analyses on overall survival. Uni- and multivariate cox regression analysis on overall survival in non-ACLD and ACLD patients.

Patient characteristics	Univariate Analysis			Multivariate Analysis		
	HR	95%CI	*p* value	HR	95%CI	*p* value
**non-ACLD (738 patients, 22 deaths (2.98%))**						
age [per year]	1.048	1.015–1.083	0.004	1.040	1.004–1.077	0.031
sex [female vs. male]	0.973	0.420–2.253	0.950	0.957	0.412–2.227	0.919
arterial hypertension	2.362	1.024–5.449	0.044	1.771	0.669–4.689	0.250
diabetes mellitus	1.164	0.344–3.934	0.807	0.678	0.173–2.663	0.578
statin indication						
- indication but no statin vs. no indication	3.895	1.403–10.818	0.009	2.938	0.905–9.536	0.073
- on statin vs. indication but no statin	0.298	0.071–1.247	0.097	0.225	0.053–0.959	0.044
BMI [per kg/m^2^]	0.946	0.868–1.031	0.205	0.936	0.853–1.026	0.158
**ACLD (527 patients, 62 deaths (11.77%))**						
age [per year]	1.071	1.044–1.099	<0.001	1.066	1.037–1.095	<0.001
sex [female vs. male]	1.104	0.659–1.848	0.707	0.986	0.577–1.685	0.959
arterial hypertension	0.017	1.117–3.023	0.017	1.518	0.872–2.641	0.140
diabetes mellitus	1.047	0.605–1.811	0.871	0.920	0.496–1.706	0.790
statin indication						
- indication but no statin vs. no indication	1.341	0.652–2.760	0.426	1.179	0.526–2.644	0.690
- on statin vs. indication but no statin	1.239	0.522–2.941	0.627	1.102	0.453–2.685	0.830
BMI [per kg/m^2^]	0.930	0.885–0.977	0.004	0.913	0.860–0.970	0.003

**Table 4:** Cox regression analyses on liver-related survival. Uni- and multivariate cox regression analysis on liver-related survival in non-ACLD and ACLD patients.

Patient characteristics	Univariate Analysis			Multivariate Analysis		
	HR	95%CI	*p* value	HR	95%CI	*p* value
**non-ACLD (738 patients, 7 liver-related deaths (0.95%))**						
age [per year]	1.161	1.074–1.254	<0.001	1.174	1.075–1.283	<0.001
sex [female vs. male]	0.473	0.092–2.437	0.371	0.453	0.081–2.519	0.365
arterial hypertension	1.800	0.403–8.051	0.442	0.858	0.146–5.051	0.865
statin indication						
- indication but no statin vs. no indication	5.568	1.019–30.420	0.047	4.094	0.629–26.658	0.140
- on statin vs. indication but no statin	0.247	0.022–2.724	0.253	0.088	0.006–1.200	0.068
BMI [per kg/m^2^]	0.923	0.785–1.089	0.335	0.943	0.751–1.185	0.615
**ACLD (527 patients, 30 liver-related deaths deaths (5.69%))**						
age [per year]	1.032	0.997–1.067	0.071	1.026	0.991–1.062	0.145
sex [female vs. male]	1.239	0.597–2.572	0.566	1.203	0.571–2.537	0.627
arterial hypertension	1.613	0.787–3.304	0.192	1.780	0.808–3.921	0.152
diabetes mellitus	0.933	0.416–2.097	0.868	1.012	0.404–2.538	0.979
statin indication						
- indication but no statin vs. no indication	1.073	0.371–3.104	0.897	1.020	0.312–3.335	0.975
- on statin vs indication but no statin	0.699	0.156–3.123	0.639	0.605	0.133–2.761	0.517
BMI [per kg/m^2^]	0.922	0.857–0.991	0.028	0.912	0.841–0.989	0.025

### Statin therapy independently improves overall survival in non-ACLD but not ACLD patients

As several studies have shown a survival benefit regarding ASCVD-related death^6^, we explored whether guideline-conform statin therapy independently influences outcomes in a “real-world” setting, as suggested by recent literature^12^ (Table 3, Table 4). To adjust for cardiovascular and established risk factors, a multivariate cox regression analysis was performed including age, sex, the presence of arterial hypertension, diabetes mellitus and body mass index (BMI).

Notably, in the uni- and multivariate cox regression analysis on liver-related mortality in non-ACLD patients, presence of diabetes mellitus was not evaluated as an independent risk factor due to the small sample size in this subgroup.

In non-ACLD patients, age and statin therapy (on statin vs. indication but no statin) independently influenced overall survival while age and BMI independently influenced overall survival in ACLD patients (Table 3).

When liver-related survival was analyzed, age independently influenced liver-related survival in non-ACLD patients while statin therapy showed a trend towards improved survival. Importantly, the absolute numbers of liver-related events in the non-ACLD setting were low. In ACLD patients, only BMI significantly influenced liver-related survival (Table 4).

To evaluate dose-dependent effects on liver-related, ASCVD-related and “other” death, statin therapy was subdivided to “low-intensity”, “moderate-intensity” and “high-intensity” statin therapy groups (classified according to the NICE guidelines^27^) and integrated as discrete variable for competing risk analyses. Results are presented in Supplementary Table S1. Statin intensity influenced liver-related survival in non-ACLD patients (SHR 0.636, 95%CI 0.474–0.854, p = 0.003) but not ACLD patients (SHR 0.836, 95%CI 0.568–1.23, p = 0.360).

To account for exposure time in the “on statin” subgroup of patients, patients entered the statin intensity analysis at the first validated time point of statin initiation. Although there was no difference in overall survival (Supplementary Fig. S3a, log-rank p = 0.213) or ASCVD-related survival (Supplementary Fig. S3c, log-rank p = 0.735), statin intensity significantly influenced liver-related survival. Patients with low intensity statin therapy showed worst liver-related survival while patients with moderate and high intensity statin therapy had improved survival rates (Supplementary Fig. S3b, log-rank p = 0.018), although absolute number of events was small.

## Discussion

While the beneficial effects of statins in patients at risk for ASCVD are well-established, there is limited evidence on their impact on dyslipidemia in patients with (advanced) chronic liver disease. This fact is also underlined by the 2013 ACC/AHA guidelines that do not discriminate patients with or without ACLD in their recommendations for statin use^3^. Although hepatotoxicity occurs only in a minority of patients^28^, there are still prevailing concerns and low rates of statin initiation in patients with chronic liver disease^29^. While data on the use of statins are available for patients with NAFLD^21^, less evidence is available for liver diseases of other etiologies. We could show that the severity of dyslipidemia differs according to the underlying liver disease, which is in line with a recently published review^4^. However, dyslipidemia “patterns” remained similar between patients with and without ACLD although, overall, cholesterol and LDL levels decreased with progression to ACLD. Finally, we found considerable underutilization of statins, indicating the necessity for increased awareness for lipid-lowering therapy in patients with CLD.

Initiation of statins should occur in early stages of liver disease, as progression to ACLD “improves” the lipid status and correlation of dyslipidemia and ASCVD becomes weaker. These findings are supported by a recently published study in patients undergoing liver transplant evaluation where lipid profiles did not differ between patients with or without coronary artery disease as evaluated by coronary angiography^30^. After Abraldes *et al*. showed in 2009 a beneficial statin effect on portal hypertension^12^, several other studies have assessed the effects of statins in patients with liver disease^9^. Interestingly, Abraldes *et al*. reported significantly decreased (total) cholesterol and triglyceride levels after 30 days, but no specific LDL-C changes^12^. In a subsequent multicenter, double-blind parallel trial, standard of care prophylaxis of variceal rebleeding was compared to standard of care prophylaxis plus simvastatin^31^. The primary end point of this study - defined as rebleeding or death – was not significantly different between groups (p = 0.423). However, the addition of simvastatin to standard of care rebleeding prophylaxis was associated with significantly decreased mortality (relative risk reduction 61%). In a preplanned subgroup analysis, mortality was significantly decreased in Child-Pugh A/B patients but not in Child-Pugh C patients. This is in line with our study showing that significant benefits related to statins use on overall and liver-related mortality were only found in non-ACLD patients.

In other studies, survival benefits with statin therapy were observed in patients with alcoholic liver disease^32^ and hepatitis B^18^. However, in the latter studies the ICD classification system was used to identify patients and no stratification of disease severity based on elastography or histology was performed. Moreover, no data on dyslipidemia or cardiovascular risk – essential for the evaluation of the indication for a statin - were reported. In addition, ASCVD risk is not only altered by cholesterol levels but also by other components of the metabolic syndrome^33^. It has to be underlined that in most studies that evaluated the effects of statins in patients with liver disease, the outcome of “hepatic decompensation” was analyzed without data on lipid levels and specific ASCVD risk profiles. In line with this, a systematic review and meta-analysis by Kim *et al*. found a significant decrease in hepatic decompensation and mortality in patients with cirrhosis^9^ – but lacks data on the impact of lipid levels and of ASCVD risk profile.

Noteworthy, our study is of retrospective nature and therefore, the exact statin initiation date as well as patient’s compliance could not be assessed in all cases. Although we have included a landmark analysis (Suppl. Fig. S3) and could determine the initiation date in most patients, some patients entered the analysis at the date of the earliest known statin exposure rather than the exact statin initiation date, which represents a limitation. Moreover, patients in the “indication but no statin” group would have required statin therapy according to the ACC/AHA guidelines but did not receive the indicated therapy. Although we have reliably verified that these patients did not take any lipid-lowering therapy, we could not reliably assess the specific reasons for withholding statin therapy in all patients. Although none of the patients was exposed to statins before and had to stop due to adverse effects to our knowledge, we cannot completely rule out the possibility of non-documented statin prescription and subsequent discontinuation (e.g. by a general practitioner) outside of our center.

Nevertheless, our  study adds novel data to the available evidence as concomitant risk factors are presented and well-adjusted for in the cox regression analysis. Additionally, our data suggest that statin treatment should be initiated early, before ACLD develops, since the associated benefits on liver-related or overall survival was limited to non-ACLD patients. Notably, different etiologies were pooled for survival analysis in non-ACLD and ACLD cohorts as there would have been an inadequate sample size in some subgroups of patients with rare liver disease etiologies. Future (multicenter) studies should therefore focus on well characterized CLD patient cohorts stratified by disease severity and investigate the impact of statin therapy on overall, liver-related and ASCVD-related survival in distinct etiologies.

Although we present a large sample size, our study has some limitations. First, despite thorough work-up of electronical medical records, validation of mortality data by the national death registry, and exclusion of patients with insufficient follow-up, the study is of retrospective nature. Therefore, there was no standardized clinical follow-up and statin intake and compliance could not be monitored.

Secondly, baseline characteristics were different between groups. While we have addressed these differences by adjusting the data analyses in a multivariate model, no adjustments could be performed for the log-rank analyses.

Finally, we could not reliably calculate the 10 years atherosclerotic cardiovascular disease risk, as we could not reliably assess smoking status in some patients. Therefore, “true” statin underutilization rates might still be underestimated in our study.

In conclusion, dyslipidemia is highly prevalent across different etiologies of liver disease – both in patients with non-ACLD as well as with ACLD. This calls for action, since statin use was associated with an improved overall and a strong trend towards improved liver-related survival in non-ACLD patients. Nevertheless, more studies are needed to evaluate the effects of statins in various etiologies of chronic liver disease – especially in the ACLD setting.

## Methods

### Study design and patient selection

All patients undergoing liver stiffness measurement (LSM) with additional controlled attenuation parameter (CAP) at the Medical University of Vienna between 01/2013 and 10/2016 were evaluated (Fig. 1a).

Of an initial database containing 3559 LSM, only patients with valid LSM results and data available on sex, age, BMI, diabetes mellitus, total cholesterol, LDL cholesterol, etiology of liver disease, information on statin prescription, arterial hypertension, stroke, myocardial infarction, peripheral artery disease, and an available follow up >24 months, were included for further analysis. Patients that had >1 LSM during follow-up were included at the time of the first LSM. Finally, 1265 patients with known etiology of liver disease were included in this retrospective analysis.

### Assessment of baseline characteristics and underlying liver disease

Baseline characteristics were evaluated at the time of LSM. Etiology of liver disease was assessed by individual chart-review. We distinguished 5 groups of patients with different etiologies: (i) fatty liver disease, due to alcohol, non-alcoholic or metabolic liver injury was referred to as (N)AFLD; (ii) hepatitis C; (iii) hepatitis B; (iv) primary sclerosing cholangitis (PSC) and primary biliary cholangitis (PBC) were grouped as “cholestatic liver disease”; (v) all other liver diseases are grouped as “other liver disease”. “Other liver diseases” included alpha-1-antitrypsin deficiency (n = 32), autoimmune hepatitis (n = 24), Budd-Chiari syndrome (n = 1), cystic fibrosis (n = 22), hemochromatosis (n = 11), Wilson’s disease (n = 5), cryptogenic liver disease (n = 31). After thorough evaluation, a total of n = 127 patients that were referred from outside the hospital showed steatosis on abdominal US but had normal BMI and normal CAP without diagnosis of liver disease. Therefore, these patients were regularly followed up and assigned to the “suspected NAFLD” rather than the “(N)AFLD” cohort and analysed as “other LD”.

### Assessment of liver fibrosis and hepatic steatosis

Liver stiffness measurement (LSM) was performed by experienced operators by transient elastography (TE) with controlled attenuation parameter (CAP) using the FibroScan^®^ (EchoSens, Paris, France) device, as previously described^34^. Overnight fasting was a prerequisite for TE measurements and a total number of 10 valid measurements was required^35^. A cutoff value of ≥10kPa defined advanced chronic liver disease (ACLD)^36^.

### Indication for statin use

The indication for statin use according to the 2013 ACC/AHA dyslipidemia guidelines was assessed^3^. In brief, patients with clinical ASCVD, LDL > 190 mg/dL or diabetes mellitus with age of 40–75 years and serum LDL levels of 70–189 mg/dL but without any prior hepatic decompensation were considered to have an indication for statin therapy (Fig. 1b). Prior decompensating events (ascites and its complications, variceal bleeding and hepatic encephalopathy) were recorded at the time of LSM, as statins are contraindicated in decompensated patients. Due to missing smoking status, the statin indication for reduction of elevated 10-year ASCVD risk could not be assessed. Figure 1b illustrates the main cohorts used for further analysis.

All data on statin use was derived from electronic medical records. During follow-up, none of the patients had intermittent statin use.

To evaluate the effect of statin-intensity on liver-related survival, we classified patients as being on “low intensity”, “moderate intensity” or “high intensity” statin therapy, according to the NICE guidelines^27^. In six patients, no information on the dosing of the prescribed statin was available despite chart review. Therefore, these patients were excluded from the competing risk sub-analysis of liver-related, ASCVD-related or other cause of death. During follow-up, two patients were discontinued from statin therapy in the high-intensity group but were analyzed as “high-intensity” patients, as they were on high-intensity statins for the majority of follow-up.

### Evaluation of concomitant metabolic disorders

To evaluate concomitant ASCVD and, therefore, additional ASCVD risk factors, arterial hypertension as well as antihypertensive medication use were assessed. Notably, non-selective betablocker therapy that was solely prescribed for treatment of portal hypertension was not considered as antihypertensive medication. Moreover, diabetes mellitus was recorded and classified as insulin dependent (IDDM) or non-insulin-dependent (NIDDM).

### Assessment of overall patient survival

In general, patients entered the analysis at the time of LSM. For landmark analysis presented in Supplementary Fig. S3, patients entered the analysis at the time of statin initiation.

To assess the overall survival after LSM, electronic medical records were retrospectively evaluated for in-hospital deaths. Additionally, the national death registry was used to record all deaths occurring outside our hospital. In the majority of patients that died during follow-up, autopsy reports were available that allowed to asses for liver-related, ASCVD related or “other” cause of death. In patients without available autopsy report, the Austrian census bureau provided the cause of death as officially registered in the national death registry. Finally, the cause of death could be verified in all patients as by national death registry query. Notably, the national death registry does record “liver-related death” but not the specific liver-related complication leading to death (e.g. variceal bleeding).

### Statistical analysis

Differences in proportions between groups were evaluated using Chi-Square or Fisher’s Exact tests whenever appropriate. To test for normal distribution, the D’Agostino’s K² test was utilized. For numerical variables and comparisons between two groups, Student’s t-test or Mann-Whitney U test was used, as applicable.

To compare overall survival between groups, Kaplan-Meier curves were used to visualize the data and the log-rank test was used to compare groups.

To evaluate the impact of statin therapy on overall and liver-related survival, uni- and multivariate cox regression analysis was utilized.

To evaluate impact of statin intensity on liver-related, ASCVD-related and other death, a competing risk analysis according to Fine and Gray^37,38^ was used. Lost to follow-up, live-related death, ASCVD-related death and other death were considered as competing events, while liver-related death, ASCVD-related or other death were the event of interest, depending on the subanalysis. The multivariate model was also used to evaluate age, sex, the presence of arterial hypertension, the presence of diabetes mellitus and BMI as covariate risk factors, next to statin intensity. Patients entered the study at the day of LSM and were censored at the time of death or the end of follow-up.

GraphPad Prism Version 8 (GraphPad Software, La Jolla, California, USA) was used for most data visualization and D’Agostino’s K² test.

The R language for statistical computing, necessary libraries^39,40^ and in particular the cmprsk library^41^ were utilized for the competing risk analysis and plotting cumulative incidence graphs.

SPSS Version 24 (IBM, New York, USA) was used for all other statistical analyses. A p-value < 0.05 denoted statistical significance.

### Institutional review board

The retrospective cohort study, including assessment of overall survival, was conducted according to the Declaration of Helsinki, and was approved by the Medical University of Vienna’s institutional review board (EK-Nr. 2013/2016; https://ekmeduniwien.at/core/catalog/2016/). The requirement of written informed consent was waived by the institutional review board.

## Supplementary information

Supplementary Figures/Tables

## Data Availability

The datasets generated and/or analyzed in the current study are not publicly available due to patient privacy but are available from the corresponding author on reasonable request. However, all relevant data are within the paper and its supporting Information files.
