# Supplementary material for: Guideline-conform statin use reduces overall mortality in patients with compensated liver disease
Source: Sci Rep. 2019 Aug 12;9:11674. doi: 10.1038/s41598-019-47943-6 (PMC6690990; doi:10.1038/s41598-019-47943-6)
Supplement: Supplementary file 1 — Supplementary Figures/Tables [file 41598_2019_47943_MOESM1_ESM.docx]

**SUPPLEMENTARY INFORMATION**

**Guideline-conform statin use reduces overall mortality in patients with compensated liver disease**

By

Lukas W. Unger, Bernadette Forstner, Stephan Schneglberger, Moritz Muckenhuber, Ernst Eigenbauer, David Bauer, Bernhard Scheiner, Mattias Mandorfer, Michael Trauner, and Thomas Reiberger



**Supplementary Figure-S1:** Prevalence of metabolic comorbidities in (a) non-ACLD patients versus (b) ACLD patients according to statin therapy. ** indicates p<0.010


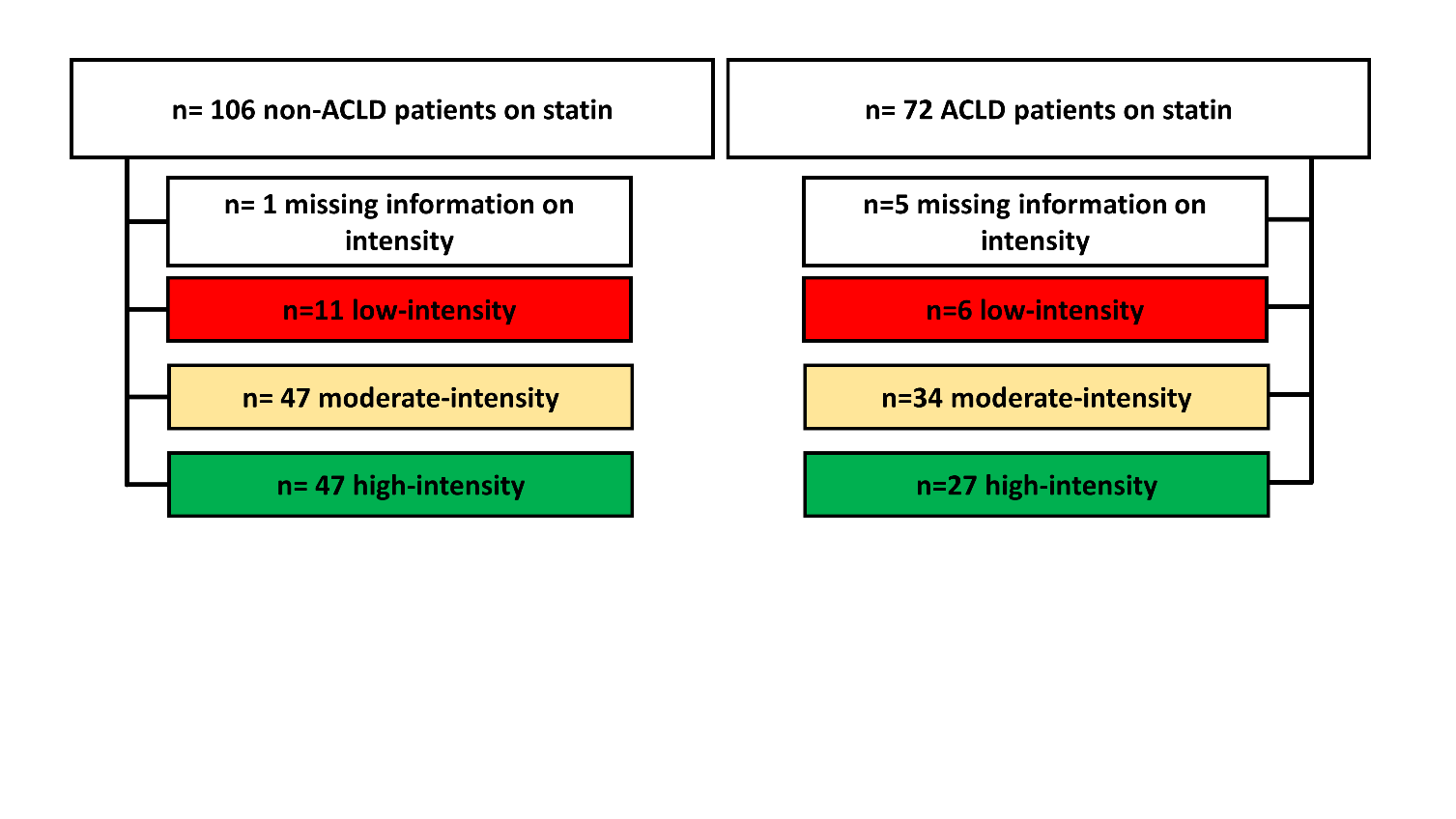
**Supplementary Figure-S2:** Statin intensity subgroups.

**Supplementary Figure-S3:** Landmark analysis of patients on statin therapy. Patients were included in the analysis at the time of statin therapy initiation.

**Supplementary Table-S1:** Competing risk analyses. (A) and (B): Subdistribution hazards for liver-related death in non-ACLD and ACLD patients (C) and (D): Subdistribution hazards for ASCVD related death in non-ACLD and ACLD patients. (E) and (F): Subdistribution hazards for other causes of death in non-ACLD and ACLD patients.

**Table-S1A: Uni- and multivariate competing risk analysis for liver-related death in non-ACLD patients**

| **patient characteristics** | **Univariate Analysis** | | | **Multivariate Analysis** | | |
| --- | --- | --- | --- | --- | --- | --- |
|  | **SHR** | **95%CI** | ***p-*value** | **SHR** | **95%CI** | ***p-*value** |
| age [per year] | 1.16 | 1.07 - 1.26 | 0.00049 | 1.177 | 1.084-1.279 | *0.001* |
| sex [F vs. M] | 2.14 | 0.417 - 10.9 | 0.36 | 2.542 | 0.532-12.132 | 0.240 |
| arterial hypertension | 1.77 | 0.405 - 7.78 | 0.45 | 0.896 | 0.253-3.171 | 0.860 |
| diabetes mellitus | n/a | n/a | n/a | n/a | n/a | n/a |
| statin intensity | 1.02 | 0.709 - 1.46 | 0.93 | 0.636 | 0.474-0.854 | *0.003* |
| BMI | 0.925 | 0.821 - 1.04 | 0.2 | 0.917 | 0.754-1.116 | 0.390 |

**Table-S1B: Uni- and multivariate competing risk analysis for liver-related death in ACLD patients**

| **patient characteristics** | **Univariate Analysis** | | | **Multivariate Analysis** | | |
| --- | --- | --- | --- | --- | --- | --- |
|  | **SHR** | **95%CI** | ***p-*value** | **SHR** | **95%CI** | ***p-*value** |
| age [per year] | 1.03 | 0.995 - 1.06 | 0.099 | 1.021 | 0.990 - 1.05 | 0.180 |
| sex [F vs. M] | 0.8 | 0.388 - 1.65 | 0.55 | 0.808 | 0.389 - 1.68 | 0.570 |
| arterial hypertension | 1.56 | 0.761 - 3.19 | 0.23 | 1.795 | 0.776 - 4.15 | 0.170 |
| diabetes mellitus | 0.92 | 0.41 - 2.06 | 0.84 | 1.052 | 0.393 - 2.82 | 0.920 |
| statin intensity | 0.903 | 0.642 - 1.27 | 0.56 | 0.836 | 0.568 - 1.23 | 0.360 |
| BMI | 0.924 | 0.847 - 1.01 | 0.075 | 0.917 | 0.828 - 1.02 | 0.095 |

**Table-S1C: Multivariate competing risk analysis for ASCVD-related death in non-ACLD patients**

| **patient characteristics** | **Multivariate Analysis** | | |
| --- | --- | --- | --- |
|  | **SHR** | **95%CI** | ***p-*value** |
| age [per year] | 1.005 | 0.888-1.140 | 0.940 |
| sex [F vs. M] | 0.927 | 0.083-10.380 | 0.950 |
| arterial hypertension | 2.068 | 0.319-13.410 | 0.450 |
| statin intensity | 0.876 | 0.315-2.440 | 0.800 |
| BMI | 1.044 | 0.807-1.350 | 0.740 |

**Table-S1D: Multivariate competing risk analysis for ASCVD-related death in ACLD patients**

| **patient characteristics** | **Multivariate Analysis** | | |
| --- | --- | --- | --- |
|  | **SHR** | **95%CI** | ***p-*value** |
| age [per year] | 1.173 | 1.094-1.260 | <0.001 |
| sex [F vs. M] | 2.089 | 0.486-8.980 | 0.320 |
| arterial hypertension | 1.312 | 0.317-5.440 | 0.710 |
| diabetes mellitus | 0.704 | 0.245-2.030 | 0.520 |
| statin intensity | 1.338 | 0.840-2.130 | 0.220 |
| BMI | 0.971 | 0.826-1.140 | 0.730 |

**Table-S1E: Multivariate competing risk analysis for other death in non-ACLD patients**

| **patient characteristics** | **Multivariate Analysis** | | |
| --- | --- | --- | --- |
|  | **SHR** | **95%CI** | ***p-*value** |
| age [per year] | 0.999 | 0.940-1.060 | 0.980 |
| sex [F vs. M] | 0.708 | 0.214-2.340 | 0.570 |
| arterial hypertension | 4.442 | 0.728-27.090 | 0.110 |
| statin intensity | 0.619 | 0.299-1.280 | 0.200 |
| BMI | 0.878 | 0.764-1.010 | 0.068 |

**Table-S1F: Multivariate competing risk analysis for other death in ACLD patients**

| **patient characteristics** | **Multivariate Analysis** | | |
| --- | --- | --- | --- |
|  | **SHR** | **95%CI** | ***p-*value** |
| age [per year] | 1.063 | 1.006-1.123 | 0.031 |
| sex [F vs. M] | 1.036 | 0.318-3.368 | 0.950 |
| arterial hypertension | 1.621 | 0.589-4.460 | 0.350 |
| diabetes mellitus | 1.161 | 0.387-3.485 | 0.790 |
| statin intensity | 0.902 | 0.434-1.874 | 0.780 |
| BMI | 0.889 | 0.802-0.986 | 0.026 |
